# A patient-reported pressure ulcer health-related quality of life instrument for use in prevention trials (PU-QOL-P): psychometric evaluation

**DOI:** 10.1186/s12955-018-1049-x

**Published:** 2018-12-10

**Authors:** Claudia Rutherford, Julia M. Brown, Isabelle Smith, Elizabeth McGinnis, Lyn Wilson, Rachael Gilberts, Sarah Brown, Susanne Coleman, Howard Collier, Jane Nixon

**Affiliations:** 1 Quality of Life Office, School of Psychology University of Sydney, Level 6 North, Chris O’Brien Lifehouse C39Z, Camperdown, NSW 2006 Australia; 2 Leeds Institute of Clinical Trials Research, Clinical Trials Research Unit, University of Leeds, Leeds, LS2 9JT UK; 3 Leeds Teaching Hospitals NHS Trust, Leeds, LS9 7TF UK; 4 Mid Yorkshire Hospitals NHS Trust, Pinderfields Hospital, Wakefield, WF1 4DG UK

**Keywords:** Pressure ulcer, Quality of life, Symptoms, Validation, Psychometrics

## Abstract

**Introduction:**

Pressure ulcer-specific patient-reported outcome (PRO) instruments should be used to inform patient care and provide a strong evidence base for interventions aimed at preventing pressure ulcers. The aim was to carry out a comprehensive evaluation of the psychometric properties of a PRO instrument designed to assess symptoms and functional outcomes in patients at high-risk of developing pressure ulcers, the PU-QOL-P instrument.

**Methods:**

We modified the original PU-QOL instrument to be suitable for patients at high risk of pressure ulcer development based on feedback from patients, specialist nurses and PRO methodologists. The modified PU-QOL-P instrument was administered to a sub-set of patients participating in the PRESSURE 2 trial. Patients completed PU-QOL-P and SF12 instruments at baseline, weeks 1 and 3, and 30 days post-treatment. We undertook psychometric evaluation of the modified PU-QOL-P to test scale targeting, scaling assumptions, reliability, validity and responsiveness.

**Results:**

The analysis sample consisted of 617 patients that completed both instruments at baseline. We found that the PU-QOL-P instrument, consisting of nine PU-specific outcomes: three symptom and six function scales, meets established criteria for reliability, construct validity, and responsiveness. Internal consistency reliability was high with all scale Cronbach alpha > 0.795 (range 0.795–0.970). The factor analysis mostly supported the six-function scale structure. Scaling assumptions were satisfied; all item-total correlations above 0.30. Convergent validity was confirmed by significant correlations between hypothesized scales as expected. PU-QOL-P scales were responsive to change: mean scale scores from baseline to 30 days post-treatment were statistically significant for all scales apart the daily activities scale (effect sizes ranged from moderate to high). As expected, worse symptoms and functioning was observed in patients who had a category 1 or 2 PU compared to patients who did not have a PU.

**Conclusions:**

The PU-QOL-P provides a standardised method for assessing pressure ulcer-specific symptoms and functional outcomes for quantifying the benefits of associated interventions from the patient’s perspective. It can be used in research with adults at risk of pressure ulcer development in all UK healthcare settings.

**Electronic supplementary material:**

The online version of this article (10.1186/s12955-018-1049-x) contains supplementary material, which is available to authorized users.

## Background

Pressure ulcers (sometimes called bedsores, pressure sores or pressure injuries) are a common chronic wound defined as “localised injury to the skin and/or underlying tissue usually over a bony prominence, as a result of pressure, or pressure in combination with shear” [1]. With widespread prevalence and incidence in all health settings [2], affecting approximately 1 in 7 hospital and 1 in 20 community patients [3, 4], pressure ulcer (PUs) are a major burden to patients, carers, and healthcare systems [5, 6].

PUs can cause distressing symptoms including pain [4, 7, 8], exudate and odour and compromise all areas of patient functioning [5, 9]. Presence of symptoms and functioning impairments can have a distal effect on health-related quality of life (HRQOL) outcomes [10]. Intensive interventions for preventing and treating PUs pose additional patient burden and further affect HRQOL [9]. Additional impact on patients results from increased care burden, prolonged rehabilitation, requirement for bed-rest, and hospitalisation [5, 10]. In this clinical context, evaluating patient-reported outcomes (PROs) such as symptoms, functioning, and HRQOL is particularly important and relevant, and there is enormous potential for PROs to be integral to treatment assessment and recommendations for PUs.

The primary goal in managing patients at risk of PU development is to minimise both the intensity and duration of pressure exposure on vulnerable skin sites (i.e. bony points of immobilised people such as hips, heels and elbows), not adapted to sustained and/or excessive loading, achieved by the provision of pressure redistribution support surfaces (e.g. beds, mattresses, mattress overlays and cushions) and patient repositioning [11, 12]. Support surfaces aim to prevent PUs by relieving pressure and cushioning vulnerable parts of the body, and distributing the surface pressure more evenly. However, we know from studies [9, 13] and clinical experience that preventative interventions can directly cause patients’ pain and discomfort, impact sleep quality and limit physical function (e.g. mobility and movement) that differs between interventions, and these proximal effects can result in severe and persistent negative  effects on HRQOL. Therefore, assessment of symptoms and impacts on HRQOL of different pressure redistribution support surfaces should be considered when deciding between management options for patients receiving prevention interventions as well as during post-prevention and treatment surveillance to enable better detection and management of symptoms that impact patients’ HRQOL. This in turn will improve psychological outcomes, HRQOL, and the quality of patient care.

Our previous work has identified PROs important to people with PUs [5, 9, 10], established the need for patient-reported measures of outcomes specific to PUs [14], and developed and evaluated a PRO instrument to assess PU-specific symptoms and functioning impacts (the PU-QOL instrument, accessed at: http://medhealth.leeds.ac.uk/puqol-ques) [15]. PRO instruments can be useful tools for evaluating health changes following interventions if they are fit for purpose and accord with international standards for rigorous development [16]. Patient-based outcome assessment in PUs is in its infancy; few studies have assessed PROs and those that have done, have mostly used generic instruments [14]. Using a PU-specific PRO instrument in future research and clinical practice could help improve the evidence-base through research assessing effectiveness of PU therapies from the patients’ perspective, facilitate clinician-patient communication and shared decision making, prioritise patient outcomes and preferences, and monitor changes in outcomes during prevention, treatment, and post-interventions [17, 18].

Comparative effectiveness research requires healthcare interventions to be evaluated not only in terms of clinical outcomes, but a comprehensive evaluation should incorporate patients’ perspectives of interventions, both in terms of patients’ actual experiences (e.g. symptoms and function), and their judgments about the value of care (e.g. access to services) [16, 19]. The original PU-QOL instrument was developed for use in comparative effectiveness research with patients with PUs receiving treatment interventions [15]. Given the heterogeneity of the PU population, further work was required to ensure that the PU-QOL instrument fit the needs of all people with PUs including those with superficial PUs, as well as patients at risk of PU development [15]. To enable assessment of PROs in patients at risk of PUs receiving preventative interventions, the aim of this study was to modify the original PU-QOL instrument so that it was suitable for use in comparative effectiveness research with patients at high-risk of PU development receiving preventative interventions. Given that modifications were planned to the original version, further aims were to undertake an evaluation of the reliability and validity of the modified version, and to also undertake responsiveness testing, which has not previously been reported. Testing of psychometric properties is an ongoing process of learning more about the construct, making new predictions, and testing them, particularly when modifications are made to a previously tested PRO instrument – each supportive study serves only to strengthen validity [20]. The participants were acutely ill in-patients at high risk of PU development receiving mattresses in common use in healthcare settings and advocated in national and international guidelines [1, 21] including ‘low tech’ constant low pressure specialist foam mattresses and ‘high tech’ electrically powered mattresses which alternate pressure distribution through air filled sacs.

## Methods

### Development of the PU-QOL-prevention instrument

The original PU-QOL is a self-report instrument [15], comprising of three symptom (pain 8 items, exudate 8 items, odour 6 items) and seven function scales: four physical functioning (sleep 6 items, movement and mobility 9 items, daily activities 8 items, vitality 5 items); two psychological well-being (emotional well-being 15 items, and self-consciousness and appearance 7 items); and one social participation (9 items), plus a single item for itchiness and a single item for global HRQOL. It is intended for interview-administration [22] with patients who have any category PU, and patients rate the amount of “bother” attributed “during the past week” on a 3-point response scale (0 = not at all - 2 = a lot). Scale scores are generated by summing items and then transforming to a 0–100 scale. High scores indicate greater patient bother. The PU-QOL instrument has been validated for use with patients with PUs and is most appropriate for people with severe PUs, as demonstrated by a lack of items to represent people with little or no bother due to PUs [15].

The original PU-QOL instrument was modified to produce a prevention version (the PU-QOL-P instrument). A group of 20 experts was convened including specialist tissue viability nurses with a minimum of five years experience managing people with PUs (recruited from participating sites involved in PU-QOL evaluation [23]), consumers with past experience of having PUs (recruited via the Pressure Ulcer Research Service User Network UK [24], and senior PRO methodologists with expertise in developing and evaluating PRO instruments. The expert group were asked to review the existing PU-QOL instrument and complete a questionnaire that asked about content (e.g. how relevant and representative are the issues to people at high-risk of pressure ulcer development?); if any items were confusing, difficult to understand or needed clarification; if any items were not relevant to people at risk of PUs; and if any important issues were missing.

### Development of the PU-QOL-prevention instrument: Results from expert group review

Feedback from the experts informed modifications to the PU-QOL instrument. Specifically, we changed the question stem from: “During the past week, how much were you bothered by these feelings because of your pressure sore(s)?” to “During the past week, how much were you bothered by these feelings because of any pressure area pain, soreness or discomfort, pressure sores or treatments?”. Feedback about content that was missing resulted in: four items added to the pain scale including “feeling of altered sensation”; “dull ache”; “feeling sore”; and “loss of feeling (e.g. numbness or paralysis)”; one item added to the sleep scale (“being woken during sleep”); and a single item developed for “overall HRQOL” with response options: improved/got better, the same or worsened. Two symptom scales (exudate and odour) were considered only relevant for people with PUs and therefore we introduced a skip question (e.g. only complete if you have a PU). Two items were removed from the Daily Activities scale: “doing shopping” and “doing jobs around the house”. We also added examples to one item “Doing things that you enjoy (e.g. reading a book, watching a movie, talking on the telephone, using a computer)”. We also added examples to two items in the Malaise scale: “Feeling tired (e.g. in need of sleep or rest)” and “Feeling fatigued (extreme tiredness resulting from mental or physical exertion or illness)”. We removed “because of your sore” from item “Feeling like you have no control over your life because of your sore” in the emotional wellbeing scale. The participation scale was considered only relevant to people who were not hospitalised so was excluded. No changes were made to the Movement/mobility and Appearance and self-consciousness scales or the single item for itchiness.

### Evaluation of the PU-QOL-prevention instrument

In order to assess the psychometric properties of the PU-QOL-P instrument, we conducted a sub-analysis of all patients recruited during August 2013 – November 2016 to the PRESSURE 2 trial that had completed both the PU-QOL-P and SF12 instruments at baseline, and provided responses to scale items. This initially included all patients randomised to the trial but due to data burden, only a sub-set of patients completed the PU-QOL-P and SF12 instruments; patients were randomised to either complete HRQOL instruments (i.e. PU-QOL-P and SF12) or utility measures. A detailed description of the methods for the PRESSURE 2 trial are published elsewhere [25]. Briefly, PRESSURE 2 is a multicentre, open-label, randomised, double triangular, group sequential, parallel group trial conducted in acute secondary care hospitals, community hospitals and NHS-funded intermediate care/rehabilitation facilities in England and Scotland. ‘High-risk’ patients admitted to an in-patient facility with evidence of acute illness were randomised, in a 1:1 allocation, to receive either a high-specification foam mattress or alternating-pressure mattress in conjunction with an electric profiling bed frame. The primary objective of the trial was to compare mattresses allocation in terms of the time to developing a new Category 2 or above PU by 30 days post-treatment phase. Secondary endpoints included time to developing a new Category 1 and 3 or above PUs, time to healing of pre-existing Category 2 PUs, HRQOL, cost-effectiveness, incidence of mattress change and safety.

### PRO instruments

The PU-QOL-P instrument (described above) was administered along with a generic measure of health status, the SF12 [26]. The SF12 was chosen on the basis of evidence from a systematic review of PRO instruments for chronic wounds (including PUs) [14] and practical issues relating to the patient population. Use of the SF-36 was considered however it was decided by the project team that it was too long for use with patients with PUs (e.g. these patients are largely elderly, highly dependent, and/or with high levels of co-morbidity including acute and chronic illness). Instead, the SF-12, a short version of the SF-36, was selected to reduce respondent burden.

The SF-12 is a generic instrument that assesses health status. It includes eight domains: physical functioning, role-physical, body pain, general health, energy/fatigue, social functioning, role-emotional and mental health. A Physical Component Summary (PCS) and a Mental Component Summary (MCS) score are generated. An acute version of the SF12 is available that incorporates a 1-week recall period, which for this condition has been found to be relevant [27]. The SF12 takes 2 min to administer and has been validated for researcher-administration. Even though the SF12 has not specifically been validated for use with people with PUs, it has wide-spread use in other chronic wounds and dermatological conditions to assess changes in health status between groups; has been used with other chronic-skin wound conditions to validate their corresponding disease-specific PRO instruments; and has been validated for use with elderly people.

### Statistical analyses

**Fig. 1:**
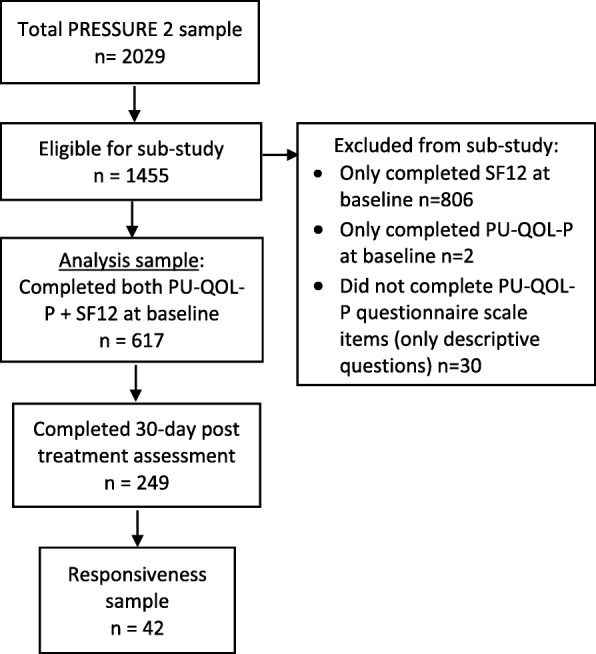
Flow of participants from the PRESSURE 2 trial included in the psychometric sub-study

Descriptive analyses were conducted to describe demographic and clinical characteristics of the patient sample. Standard psychometric analyses were used to evaluate the PU-QOL-P. Analyses were performed using SPSS® Statistics for Windows® version 22.0 (IBM, Armonk, New York, USA). All analyses were conducted with two-tailed tests at five level of significance. Analyses were performed on a sub-sample of patients from the PRESSURE 2 trial that completed both the PU-QOL-P and SF12 questionnaires at baseline (*n* = 617; Fig. 1). Responsiveness analysis was limited to patients who also completed PU-QOL-Ps at 30 days post-treatment.

### Scale-to-sample targeting and missing data

Scale-to-sample targeting was determined by investigating whether scale scores spanned the entire possible scale range and floor/ceiling effects were low (less than 20%). Missing data rates were assessed by completeness of item- and scale-level data. The minimum criterion for computable scale scores was 50%.

### Reliability

Internal consistency reliability was assessed for the eight multi-item scales using Cronbach’s α coefficient, with an α value of at least 0.8 regarded as adequate, and a value of 0.7 or more as acceptable for group comparisons [28].

### Within scale validity: Multitrait scaling

Multitrait scaling was used to evaluate the hypothesized scale structure of the PU-QOL-P. Within-scale construct validity assesses whether it is appropriate to sum pre-specified groups of items to generate a scale score that reflects a single underlying construct. Scaling assumptions are satisfied with similar item means and corrected item-total correlations (ITC) > 0.3. Corrected ITCs > 0.3 indicate that items within each scale contain a similar proportion of information. ITCs of 0.4–0.6 were considered moderate, and those exceeding 0.6 were considered high [29].

### Factor analysis

Exploratory factor analysis (EFA) was performed to determine whether the proposed nine-scale structure of the PU-QOL-P was supported in an at-risk population. Exploratory factor analysis was chosen as the testing population was very different to the population in which the original PU-QOL instrument was tested in, and a factor analysis had not previously been undertaken. Direct Oblimin rotation methods allowed principal axis factoring extraction deducing factor correlations. The suitability of the data for EFA was assessed using the Kaiser–Myer–Olkin measure of sampling adequacy (0.8) and Bartlett’s test of sphericity (χ2 less than 0.01). EFA contributes evidence towards construct validity.

### Construct validity: Between scales validity

Convergent validity and discriminant validity were assessed at the item level within the multitrait scaling (e.g. convergent validity was confirmed when an item correlated highly with the scale; ITC > 0.3), and at the scale level using Spearman *ρ* rank-order correlation coefficients between conceptually related scales of PU-QOL-P and SF12. It was hypothesized that conceptually related scales would correlate more highly than unrelated scales (high, *r* > 0.7; moderate, r 0.3–0.7; low, *r* < 0.3). These criteria were used as guides to the magnitude of correlations, as opposed to pass–fail benchmarks. As the PU-QOL-P assesses condition-specific issues and the SF12 generic health issues, moderate correlations were expected between the PU-QOL-P scales for pain, mobility and movement, daily activities, malaise, and emotional well-being with related scales of the SF12.

### Known-groups validity

Another aspect of construct validity, known-groups, evaluated the extent to which PU-QOL-P scales differentiate between groups of patients defined by clinical criteria. We had difficulty devising clinical groups as the literature proved little evidence towards clinical groups known to differ. However, based on clinical experience, we hypothesized that the PU-QOL-P scales, particularly function scales, would differentiate between presence of category 2 PU (no versus yes category 2 PU at baseline); Braden score [30](completely limited versus no/slight impairment); and adverse events [25] (AEs; no versus yes AEs at 30 days post-treatment). As exploratory analyses, it was tentatively hypothesized that the PU-QOL-P scales might differentiate between presence of category 1 PU (no versus yes category 1 PU at baseline) and PU location (torso versus limb sites).

Each of the hypotheses was tested with an independent-samples t-test, and a corresponding effect size (ES) was calculated to indicate the size of the effect as follows: small (0.20–0.49), moderate (0.50–0.79) or large (0.80 or more) [31, 32].

### Responsiveness-to-change analysis

Responsiveness can be considered longitudinal validity, and analyses should be conducted in data where clinically important change is expected. A sub-set of patients with PUs at baseline that healed by 30 days post-treatment,  and patients with no PU at baseline that developed one by 30 days post-treatment provided such data. Paired sample t-test was used to evaluate the significance of score changes between these two time points, with expected significant changes in both symptom and function scales in patients who have category 1 or 2 PUs at baseline but no PU at 30 days post-treatment. Corresponding effect size values were calculated as the mean change from baseline to 30 days post-treatment, divided by the standard deviation of change [31–33].

## Results

### Evaluation of the PU-QOL-prevention instrument

#### Baseline demographics

No notable differences were observed between the analysis sample, who completed both PU-QOL-P and SF12 instruments, and those excluded from the analysis (i.e. those who only completed SF12) except that the analysis sample included a few more people with category 2 PUs. The analysis sample consisted of 617 patients (45.1% male), aged between 21.9 to 101.3 years (mean 75.7), of which 141 (22.8%) had a category 1 or 2 PU and 61.4% reported presence of pressure-related pain at baseline. The majority (99.8%) were Caucasian. More than half (65.6%) were from secondary care hospital setting with a medical condition (58.8%), and 70.7% were considered as having  very limited mobility according to the Braden scale.

#### Scale-to-sample targeting and missing data

Scale scores spanned the entire scale ranges for all scales apart from exudate (0–86) and odour (0–25) scales. Global QOL mean score was near the scale midpoint, however, all other mean scale scores were below 37, with all scales exceeding the 20% criterion for floor effects. Scale scores were computable for over 70% of respondents (range 73.8–100%) (see Additional file 1).

#### Reliability

Internal consistency reliability was high with all scale Cronbach coefficient alpha > 0.795 (range 0.795–0.970; Table 1). During the analysis, items ‘putrid smell’ and ‘sickening smell’ were removed from the odour scale because they had zero variance therefore the results are based on a four not six item scale.

**Table 1:** PU-QOL-P scale level analyses - Reliability and scaling assumptions: Validity within-scale analysis

PU-QOL-P scale (n items)	Internal consistency		Mean inter-item correlation	Inter-item correlation^a^	Scaling Assumptions -Corrected ITC^a^
	N^c^	Cronbach’s alpha			
Pain (12)	617	0.864	0.355	0.128–0.713	0.364–0.642
Exudate (8)	60^b^	0.860	0.451	−0.024 – 0.865	0.337–0.824
Odour (6)	61^b^	0.795	0.492	−0.017 – 1.000	− 0.017 – 0.893
Sleep (7)	333	0.937	0.682	0.540–0.827	0.753–0.871
Movement & mobility (9)	177	0.963	0.744	0.593–0.928	0.803–0.909
Daily activities (6)	186	0.937	0.705	0.476–0.900	0.581–0.914
Malaise (5)	215	0.915	0.680	0.559–0.844	0.669–0.846
Emotional well-being (15)	220	0.970	0.681	0.447–0.900	0.630–0.869
Self-consciousness and appearance (7)	305	0.921	0.645	0.532–0.786	0.695–0.818

*SEM* standard error mean; *IIC* inter-item correlation; *ITC* item-total correlation (corrected for overlap)

^a^Range

^b^sample *n* = 62; only patients with a category ≥2 PU complete exudate and odour scale

^c^n estimated based on listwise deletion; observations that have one or more missing values across all variables are removed from analysis

#### Factor analysis

We were unable to include the symptom scale items into the factor analysis as there were fewer than two people, at least one of the variables had zero variance, there was only one variable in the analysis, or correlation coefficients could not be computed for all pairs of variables. Conceptually, it made sense to consider the items within our six function scales separately from the symptom items.

The suitability of data for EFA was assessed. Kaiser–Myer–Olkin value was 0.530; slightly below the recommended value of 0.6 or above, but the Bartlett’s test of sphericity reached statistical significance (*χ*2=10,159.415, *ρ* 0.000). Inspection of the correlation matrix revealed the presence of many correlation coefficients above 0.30 (all but one item), suggesting the EFA results could be considered [34]. The EFA revealed the presence of five components with eigenvalues exceeding 1, explaining 26.52, 4.24, 2.31, 1.63, and 1.36 of the variance respectively. The five component solution explained a total of 73.59% of the variance.

The EFA (see Additional file 2) mostly supported a six-function scale structure. Items with a factor-loading coefficient ≥ 0.4 in each factor (scale) were considered against our hypothesised scale structure. Factor one included all items from the emotional wellbeing scale, apart from one item “Feeling that people avoided you or treated you differently now”, which loaded with items in the appearance and self-consciousness scale, and the addition of item “feeling helpless” from the appearance/self-consciousness scale. All items from the appearance/self-consciousness scale loaded together in factor five except item “feeling helpless” and the addition of item “Feeling that people avoided you or treated you differently now”.

Factor two included items from the movement and daily activities scales. It is not surprising that movement and daily activity items would group together into one factor given that having reduced mobility would correlate highly with reduced ability to participate in daily activities; both scales assess aspects of physical function. Item “Being emotionally close or affectionate with loved ones (e.g. able to cuddle, being intimate)” from the activity scale had weak item-factor weights (< 0.3) across all factors. Inspection of missing item rates revealed that the intimacy item was not answered by 12.2% of the sample at baseline and by 25.5% of the sample at 30 days post-treatment. Factor three included all sleep items. Factor four included all malaise items except item “Feeling that your appetite has reduced”.

#### Within scale validity

Scaling assumptions were satisfied (Table 1). Mean inter-item correlations for all scales ranged 0.355–0.744. All item–own-scale correlations were moderate to high (ITC; all > 0.45) for the six function scales but not the three symptom scales. Corrected ITCs were above 0.30 (range 0.337–0.803), satisfying recommended criteria (> 0.3), except for the odour scale (corrected ITC range 0.02–0.89).

#### Between scale validity

Correlations between PU-QOL-P and SF12 scales were generally low to moderate (Table 2), suggesting that PU-QOL-P scales provide distinct constructs (i.e. disease-specific outcomes) from those measured by the SF12. Convergent validity was confirmed by significant correlations between hypothesized scales as expected. As predicted, PU-QOL-P mobility scale correlated significantly with SF12 physical function and role physical scales. Also as expected the PU-QOL-P sleep scale, correlated significantly with SF12 vitality scale, but unexpectedly the malaise scale did not. PU-QOL-P emotional wellbeing and self-consciousness/appearance scales correlated significantly with SF12 role emotional and mental health scales. PU-QOL-P pain scale correlated significantly with the SF12 pain scale (Table 2).

**Table 2:** Spearman’s rho correlations between PUQOL-R and SF12 scales (convergent validity)

Scale (n items)	Physical function (2)	Role physical (2)	Bodily pain (1)	General health (1)	Vitality (1)	Social function (1)	Role emotional (2)	Mental health (2)
Pain (12)	0.072	0.109^a^	0.320^a^	0.220^a^	0.168^a^	0.046	0.201^a^	0.261^a^
Exudate (8)	0.005	0.112	0.036	0.046	0.237	0.183	0.229	0.279^b^
Odour (6)	0.118	0.235	0.109	0.149	0.022	0.154	0.160	0.063
Sleep (7)	0.143^a^	0.137^a^	0.283^a^	0.203^a^	0.110^b^	0.008	0.246^a^	0.282^a^
Movement & mobility (9)	0.151^a^	0.133^a^	0.350^a^	0.214^a^	0.171^a^	0.036	0.299^a^	0.300^a^
Daily activities (6)	0.086	0.071	0.197^a^	0.139^a^	0.027	0.046	0.238^a^	0.218^a^
Malaise (5)	0.030	0.017	0.229^a^	0.205^a^	0.024	0.076	0.293^a^	0.274^a^
Emotional well-being (15)	0.034	0.037	0.227^a^	0.179^a^	0.012	0.030	0.323^a^	0.275^a^
Self-consciousness and appearance (7)	0.034	0.053	0.219^a^	0.119^a^	0.053	0.045	0.249^a^	0.206^a^
Itchiness (1)	0.006	0.080	0.130^a^	0.071	0.033	0.030	0.100^b^	0.100^b^

^a^Correlation is significant at the 0.01 level (2-tailed)

^b^Correlation is significant at the 0.05 level (2-tailed)

### Known groups validity

#### Presence of category 2 PU (no versus yes category 2 PU at baseline)

Known-group comparisons were not found to be statistically significant for no versus yes category 2 PU at baseline groups. However, small to moderate effect size values were observed for all scales (see Additional file 3). As expected, all scales apart from the self-consciousness scale had higher mean scores in the category 2 PU group compared to the no category 2 PU group. Mean differences in scores ranged from 1.28 to 9.52 (see Additional file 3). Higher scores indicate worse symptom burden or impaired function.

### Braden score (completely limited versus no/slight impairment)

All mean scores were higher for the completely limited group compared to the no/slightly impaired group in all six PU-QOL-P function scales (see Additional file 3). As expected, we found significant differences between completely impaired (M = 49.38, SD = 44.6) and no/slightly impaired (M = 28.72, SD = 33.0) groups for the mobility scale, *p* = 0.013 and the effect size was moderate (0.60); and between completely impaired (M = 35.56, SD = 43.2) and no/slightly impaired (M = 14.14, SD = 26.5) groups for the daily activities scale, *p* = 0.007 and the effect size was moderate (0.75).

### Exploratory known groups included PU location (torso versus limb sites)

Higher mean scores were observed in the three symptom and the mobility scales in people with torso PUs compared to those with limb PUs; effect sizes were small (see Additional file 3). However, those with limb PUs reported higher (or worse) mean scores in sleep, daily activities, malaise, emotional wellbeing, and self-consciousness scales compared to those who had torso PUs.

It is important to note that for all known groups we had small samples (range 2–31 patients) therefore known groups results are considered preliminary.

### Responsiveness to change

In patients who had a category 1 or 2 PU at baseline that healed by 30 days post-treatment, PU-QOL-P mean scale scores from baseline to 30 days post-treatment were statistically significant (*P* < 0.001) for scales pain (effect size (ES) 0.86 large), sleep (ES 0.48 moderate), malaise (ES 0.61 moderate), emotional wellbeing (ES 0.65 moderate), and appearance/self-consciousness (ES 0.60 moderate) but not the two physical function scales (Table 3). In patients who had a category 1 or 2 PU at baseline, all scales showed higher mean scores at baseline compared to at 30 days post-treatment (range mean change 4.337–18.569).

**Table 3:** PU-QOL-P Responsiveness over time: PU at baseline (visit 0) compared to no PU 30 days post-treatment end (visit 30)

Scales (n items)	N	Baseline scores	Visit30 scores	Mean change	Effect Size	CI	*P* value
		Mean (SD)	Mean (SD)				
Pain (12)	31	27.57 (25.553)	9.001 (16.927)	18.569	0.86	9.637, 27.493	0.000
Exudate (8)	0						
Odour (6)	0						
Sleep (7)	29	27.82 (32.804)	14.03 (23.277)	13.790	0.48	1.655, 25.899	0.027
Movement & mobility (9)	21	37.86 (36.769)	21.17 (33.085)	16.688	0.48	−7.811, 41.179	0.171
Daily activities (6)	25	13.67 (28.956)	9.333 (24.244)	4.337	0.16	−10.069, 18.736	0.540
Malaise (5)	23	21.09 (37.049)	3.913 (13.731)	17.177	0.61	3.705, 30.642	0.015
Emotional well-being (15)	30	10.17 (20.837)	0.476 (2.608)	9.694	0.65	1.906, 17.475	0.016
Self-consciousness and appearance (7)	28	8.67 (19.668)	0.255 (1.350)	8.415	0.60	0.803, 16.034	0.032

In patients who did not have a category 1 or 2 PU at baseline but developed one by 30 days post-treatment, movement (ES 0.28 small), activities (ES 0.45 moderate), malaise (ES 0.34 small), and self-consciousness (ES 0.18 small) scale mean scores were higher at baseline for people with no PU compared to those who developed a PU by 30 days post-treatment; Table 4) but these results were not statistically significant. This finding may in part be due to the fact that this sample was acutely ill at baseline (e.g. immobile and unwell), placing them at-risk of PUs and consequently contributed to the PU developing. All other scale scores were higher at 30 days post-treatment compared to baseline, suggesting that pain, sleep and emotional wellbeing is worse in patients with a PU compared to those without, although again the differences were not statistically significant (Table 4). These findings are preliminary due to the small sample sizes.

**Table 4:** PU-QOL-P Responsiveness over time: no PU at baseline (visit 0) compared to PU 30 days post-treatment end (visit 30)

Scales (n items)	N	Baseline scores	Visit30 scores	Mean change	Effect Size	CI	*P* value
		Mean (SD)	Mean (SD)				
Pain (12)	11	30.37 (27.522)	35.455 (34.676)	−5.085	−0.16	−22.166, 12.000	0.522
Exudate (8)	0						
Odour (6)	0						
Sleep (7)	9	17.26 (23.487)	26.243 (30.829)	−8.983	−0.33	−32.791, 14.828	0.410
Movement & mobility (9)	7	61.79 (48.055)	48.866 (44.792)	12.924	0.28	−3.955, 29.806	0.110
Daily activities (6)	7	9.64 (16.610)	3.5714 (9.449)	6.069	0.45	−4.596, 16.739	0.213
Malaise (5)	7	13.21 (24.440)	6.548 (12.697)	6.662	0.34	−8.416, 21.749	0.321
Emotional well-being (15)	11	11.31 (21.949)	18.375 (31.309)	−7.065	−0.26	−24.106, 9.985	0.378
Self-consciousness and appearance (7)	11	4.24 (9.898)	2.597 (8.615)	1.643	0.18	−7.771, 11.061	0.705

### Post-hoc analyses

The results from the psychometric analysis suggested modifications that could be made to four of the PU-QOL-P instruments’ scales. As such, we: (1) removed item “appetite” from the malaise scale; (2) removed item “intimacy” from the daily activities scale; (3) removed item “helpless” from the self-consciousness scale and added it to the emotional wellbeing scale; and (4) removed item “people treat me differently” from the emotional wellbeing scale and added it to the self-consciousness scale. These changes were considered to make sense conceptually, and re-analysis of the psychometric properties supported these modifications. Specifically, most patients were hospitalised at the time of completing questionnaires so answering a question about intimacy may have appeared irrelevant or considered too personal. Following the modifications, the internal consistency reliability and within scale construct validity was retained in all four scales, with the daily activities, malaise, and emotional wellbeing scales values for Cronbach alpha (range 0.914–0.971 for all four modified scales), inter-item correlations (all > 0.502), and corrected item-total correlations increasing (all > 0.681). Although for the self-consciousness scale the Cronbach alpha decreased marginally from 0.921 to 0.914, mean inter-item correlations decreased from 0.645 to 0.628 (range from 05.32–0.786 to 0.502–0.795), and corrected item-total correlations decreased from 0.695–0.818 to 0.681–0.812, all values remained within acceptable ranges. Convergent validity results were strengthened following the modifications. PU-QOL-P daily activity scale was significantly correlated with SF12 physical functioning and role physical scales, both PU-QOL-P psychological scales were significantly correlated with SF12 role emotional and mental health scales, and PU-QOL-P malaise was now significantly correlated with SF12 vitality scale (see Additional file 4).

### Final PU-QOL-P

The final PU-QOL-P prevention version is a researcher-administered instrument, comprising of three symptom scales: pain (12 items), exudate (8 items), odour (6 items); six function scales: four physical functioning (sleep 7 items, movement and mobility 9 items, daily activities 5 items, malaise 4 items) and two psychological wellbeing (emotional wellbeing 15 items and self-consciousness and appearance 7 items); and three single items for itchiness, appetite, and global QOL. Patients rate the amount of “bother” attributed “During the past week” on a 3-point response scale (e.g. 0 = not at all - 2 = a lot). Scale scores are generated by summing items and then transforming to a 0–100 scale. High scores indicate greater patient bother. The PU-QOL-P instrument is intended for interview-administration, following a user manual, but could be self-completed by patients depending on their preference [35]. It is suitable for use with any adults at high risk of PU development receiving preventative interventions in the acute and community healthcare settings. Scales can be selected depending on the nature of the research. For example, the exudate and odour scales are not intended for people at risk of PU development or with superficial category 1 PUs. Electronically defined ‘skip’ questions have been added to assist in selecting scales relevant to each individual’s circumstance or the exudate and odour scales could be excluded in future prevention trials. It takes around 15–20 minutes to complete in its entirety.

## Discussion

The PU field requires a strong evidence-base that incorporates assessment of PROs. To fully capture and quantify the patients’ perspective, appropriately constructed and validated PRO instruments are required. PU-QOL-P scales mostly satisfy criteria for reliability, validity, and responsiveness in line with recommended FDA guidelines for PRO instruments [16]. The item-total correlations, alpha coefficient and homogeneity coefficient (inter-item correlation mean and range) provide evidence towards the reliability and internal construct validity of the PU-QOL-P scales. The results of the factor analysis mostly supported the use of the items as hypothesised into six function scales. The weak Kaiser–Myer–Olkin value questions suitability of the data for factor analysis so of benefit would be further confirmatory factor analysis in a new sample. However, the second test to determine suitability of the data for factor analysis, the Bartlet test, was supported, and the factor structure emerged into conceptually meaningful and logical factors. We also observed low correlations between PU-QOL-P and SF12 scales hypothesised to be conceptually related. We hypothesized that conceptually related scales would correlate more highly than unrelated scales and used the standard correlation criteria as guides to the magnitude of correlations, as opposed to pass–fail benchmarks. Importantly, correlations for scales hypothesised to be conceptually related were consistently higher than for scales hypothesised to be unrelated. Importantly, the two measures assess different constructs so even though they might be theoretically related, they are not the same (i.e. SF12 is not a criterion measure of PU-specific HRQOL). Of clinical importance is a PRO instruments’ ability to detect clinical groups known to differ and to detect change when change has occurred and these aspects were supported.

Some modifications were made to four scales, which were supported by the post-hoc  analyses, and the changes made are considered conceptually sensible. For example, items ‘putrid smell’ and ‘sickening smell’ were removed from the odour scale because they assess smell aspects associated with severe PUs. Item “Feeling that your appetite has reduced” did not correlate with other items hypothesised to make up the malaise scale. Upon reflection, this item may be assessing a different construct and could be retained as a single item for appetite. As some changes were made to the scales, some might argue that a further set of data should be collected for further validation purposes. No one test confirms validity, rather validation of a PRO instrument is an ongoing process, with the accumulation of clinical validation data building a case for a particular instrument functioning effectively in a particular population for a specific purpose [36].

Our findings contribute evidence towards support that people with category 2 PUs experience worse symptoms and functioning outcomes than those without PUs. People who are also physically limited experience worse mobility outcomes and ability to participate in daily activities than those who have no or only slight physical impairment. PUs are often a secondary comorbidity and a consequence of the primary condition a patient may be experiencing. PUs contribute additional impairment in physical function outcomes beyond those caused by other comorbidities. Our exploratory hypothesis testing suggests that patients with torso PUs experience worse symptoms and more mobility problems, while patients with limb PUs have more problems with sleep quality, daily activities and malaise, lower emotional wellbeing, and feel more self-consciousness.

Due to the small sample sizes in our hypothesised known groups, we cannot make definitive conclusions. However, we observed trends in scores in the right direction even though some were not statistically significant. Small sample size affects the standard error so we might expect to see large confidence intervals, however sample size does not affect means, standard deviations or effect size. Therefore, effect sizes are still relevant and informative, even if non-significant correlations are observed; which in this case may be attributed to sample sizes being too small to detect significant differences. Given that baseline data was completed in hospital after admission for acute illness while the 30-day post-treatment assessment was usually completed after discharge, it is possible that the changed setting may have contributed to improved HRQOL outcomes over time. However, we did look at change in both directions for the responsiveness analysis (i.e. no PU at baseline compared to PU at 30 days post treatment and also PU at baseline but no PU at 30 days post treatment).

A limitation of our study was scale-to-sample targeting; mean scores were below scale mid-points and all scales exceeded the 20% criterion for floor effects. However, given that we intended to recruit an at-risk population (i.e. few people had category 1 or 2 PUs at baseline, and none had category ≥3 PU), this finding is expected. The floor effects indicate more homogeneity in the sample than is representative of the PU population. However, our study sample is representative of a high-risk PU population, who may be experiencing pressure-area related pain but that do not experience symptoms associated with severe PUs such as exudate and odour, and the PU-QOL-P version is intended for prevention trials.

The above limitations do not preclude use of the PU-QOL-P instrument. PU-QOL-P scales can be included as one outcome measure, amongst others, for group comparisons in future PU research (e.g. clinical trials). Work is underway to develop a short-form and to test its clinical utility for use in clinical practice. As the PU-QOL-P was developed and evaluated in the UK, the validity and reliability are characteristics of the instrument for a specific population (i.e. Caucasian English speaking UK nationals). A language translation or cross-cultural adaption may be required to ensure that the PU-QOL-P is appropriate for cultures, languages and ethnic groups outside the UK (see the PU-QOL-P instrument website for guidance on language translation and cross-cultural adaptation processes: https://medhealth.leeds.ac.uk/info/423/skin/1738/purpose_puqol/2.

## Conclusions

This study makes important contributions to the PU field. The PU-QOL-P instrument provides a means for the comprehensive assessment of PU-specific PROs and for quantifying the benefits and harms of PU preventative interventions from the patients perspective; thus far lacking in the area. PRO assessment needs to become more commonplace in the PU field so that the goal of PU prevention and management can be to enhance and maintain the HRQOL of people at risk of or with PUs. The PU-QOL-P is a tool with which to evaluate whether PU preventative interventions and the healthcare given achieve this; outcomes that are ultimately best judged by patients themselves.

## Additional files

Additional file 1: Baseline data for sample who completed PU-QOL-P (*n* = 617): Scale data completeness and targeting. (PDF 302 kb)

Additional file 2: PU-QOL-P function items: Exploratory factor analysis with Oblimin rotation (PDF 219 kb)

Additional file 3: Known groups: No PU at baseline vs category 2 PU at baseline (PDF 234 kb)

Additional file 4: Modified scales post hoc analysis (PDF 93 kb)

## Abbreviations

EFA: Exploratory factor analysis
ES: Effect size
FDA: USA Food and Drug Administration
HRQOL: Health-related quality of life
PROs: Patient-reported outcomes
PU: Pressure ulcer
PU-QOL-P: Pressure Ulcer Quality of Life Instrument – Prevention version
HRQOL: Health-Related Quality of Life
SF12: Short Form 12 Questionnaire
