# Supplementary material for: A patient-reported pressure ulcer health-related quality of life instrument for use in prevention trials (PU-QOL-P): psychometric evaluation
Source: Health Qual Life Outcomes. 2018 Dec 10;16:227. doi: 10.1186/s12955-018-1049-x (PMC6288857; doi:10.1186/s12955-018-1049-x)
Supplement: Supplementary file 1 — Baseline data for sample who completed PU-QOL-P (n = 617): Scale data completeness and targeting. (PDF 302 kb) [file 12955_2018_1049_MOESM1_ESM.pdf]

Additional file 1 Baseline data for sample who completed PU-QOL-P (n=617): Scale data completeness and targeting

**Scale (n items)**	**Data completeness -** Computable scale scores (%)	**Targeting**						
		**Possible score range**	**Range mid-point**	**Observed score range**	**Mean score***	**SD**	**Floor/Ceiling effect (%)**	**Skewness**
Pain (12)	617 (100)	0 – 100	50	0 – 100	20.90	24.594	37.5/0.8	1.130
Exudate (8)	62^ (100)	0 – 100	50	0-86	5.83	15.525	75.8/1.6	3.749
Odour (6)	62^ (100)	0 – 100	50	0-25	0.54	3.330	96.8/1.6	6.927
Sleep (7)	538 (87.2)	0 – 100	50	0-100	21.72	30.880	53.0/4.7	1.274
Movement & mobility (9)	449 (72.8)	0 – 100	50	0-100	36.67	38.022	42.1/12.7	0.442
Daily activities (6)	446 (72.3)	0 – 100	50	0-100	20.50	32.734	64.1/5.7	1.346
Malaise (5)	449 (72.8)	0 – 100	50	0-100	18.01	30.386	67.0/5.0	1.537
Emotional well-being (15)	514 (83.3)	0 – 100	50	0-100	15.83	27.965	63.6/2.7	1.744
Self-consciousness and appearance (7 items)	518 (83.9)	0 – 100	50	0-100	8.70	20.131	75.3/1.1	2.730
Itchiness (1)	549 (89.0)	0 – 100	50	0-100	12.28	27.496	81.3/5.8	2.165
Global QOL (1)	565 (91.6)	0 – 100	50	0-100	47.639	38.592	32.2/27.5	0.081

∗High scores indicate great bother/impact

^sample n=62; only patients with a category ≥2 PU complete exudate and odour scales.

SD standard deviation

Floor effect =% scoring 100 (greatest bother/impact); ceiling effect = % scoring 0 (least bother/impact)

Table Baseline data for sample who completed SF-12 (n=1425): Scale data completeness and targeting

**Scale (n items)**	**Data completeness -** Computable scale score (%)	**Targeting**						
		**Possible score range**	**Range mid-point**	**Observed score range**	**Mean score***	**SD**	**Floor/Ceiling effect (%)**	**Skewness**
Physical functioning (2)	1425 (100)	0 – 100	50	0 – 100	8.608	20.281	80.3/1.6	2.641
Role physical (2)	1425 (100)	0 – 100	50	0 – 100	21.497	23.653	37.7/2.0	1.207
Bodily pain (1)	1425 (100)	0 – 100	50	0 – 100	51.51	37.040	20.0/25.5	-0.003
General health (1)	1425 (100)	0 – 100	50	0 – 100	34.32	30.321	29.2/2.8	0.488
Vitality (1)	1425 (100)	0 – 100	50	0 – 100	22.52	25.636	45.1/2.2	1.043
Social functioning (1)	1425 (100)	0 – 100	50	0 – 100	47.73	41.620	33.1/30.1	0.105
Role emotional (2)	1425 (100)	0 – 100	50	0 – 100	65.41	32.008	5.3/32.9	-0.449
Mental health (2)	1425 (100)	0 – 100	50	0 – 100	56.39	25.504	3.2/5.8	-0.230
Physical component score	1356 (95.2)	0 – 100	50	10 – 59	30.55	7.700	0.1/0.1	0.259
Mental component score	1355 (95.1)	0 – 100	50	13 – 75	45.16	12.275	0.1/0.1	-0.176

*High scores indicate better health status (or less bother/impact)

SD standard deviation

Floor effect =% scoring 100 (least bother/impact); ceiling effect = % scoring 0 (greatest bother/impact)
