# Supplementary material for: A patient-reported pressure ulcer health-related quality of life instrument for use in prevention trials (PU-QOL-P): psychometric evaluation
Source: Health Qual Life Outcomes. 2018 Dec 10;16:227. doi: 10.1186/s12955-018-1049-x (PMC6288857; doi:10.1186/s12955-018-1049-x)
Supplement: Supplementary file 2 — PU-QOL-P function items: Exploratory factor analysis with Oblimin rotation (PDF 219 kb) [file 12955_2018_1049_MOESM2_ESM.pdf]

Additional file 2. PU-QOL-P function items: Exploratory factor analysis with Oblimin rotation

Item	Factors and loadings (exploratory factor analysis)[a]				
	Factor 1	Factor 2	Factor 3	Factor 4	Factor 5
EW-frustrated	.816				
EW-fed-up	.811				
EW-miserable	.792				
EW-concerned /worried	.762				
EW-annoyed/ irritated	.750				
EW-anxious	.737				
EW-no control	.726				
EW-dependent	.713				
EW-burden on others	.687				
EW-missing out	.668				
EW-cut-off/ isolated	.624				
EW-depressed	.622				
SCA-helpless	.616				
EW-lonely	.596				
EW-angry	.524				
MM-limited in using stairs		.946			
MM-walking slowed		.910			
MM-difficulty standing		.897			
MM-limited in ability to walk		.891			
MM-difficulty transferring		.796			
DA-toilet		.753			
DA-washing self		.740			
DA-dressing		.737			
DA-doing regular activities		.657			
MM-difficulty pushing up		.612			
MM-difficulty adjusting		.522	.377		
MM-difficulty turning		.521	.357		
DA-doing things you enjoy		.507			
MM-difficulty sitting		.485			
M-appetite		.332		.319	
S-kept awake			.858		
S-not getting sleep			.812		
S-woken during sleep			.797		

S-interrupted sleep			.746		
S-trouble falling asleep			.735		
S-sleep in one position			.613		
S-uncomfortable		.318	.568		
M-unwell				.493	
M-fatigued	.310			.487	
M-reduced energy		-.377		.425	
M-tired			.389	.422	
SCA-uneasy being close					.831
SCA-lack understanding from others					.750
SCA-embarrassed					.699
SCA-physically unattractive					.612
SCA-self-conscious					.549
EW-people treat you differently					.492
SCA-lack confidence	.357				.483
DA-intimacy*					

[a]Principal axis factoring extraction with a direct oblimin rotation with Kaiser Normalisation to allow factors to be correlated. Rotation converged in 13 iterations.

Only factor-loading coefficient ≥0.3 are presented.

Items with a factor-loading coefficient ≥0.4 are retained in each factor (scale).

S sleep scale; MM movement and mobility scale; DA daily activities scale; M malaise scale; EW emotional wellbeing scale; SCA self-consciousness and appearance scale
