# Supplementary material for: A patient-reported pressure ulcer health-related quality of life instrument for use in prevention trials (PU-QOL-P): psychometric evaluation
Source: Health Qual Life Outcomes. 2018 Dec 10;16:227. doi: 10.1186/s12955-018-1049-x (PMC6288857; doi:10.1186/s12955-018-1049-x)
Supplement: Supplementary file 3 — Known groups: No PU at baseline vs category 2 PU at baseline (PDF 234 kb) [file 12955_2018_1049_MOESM3_ESM.pdf]

**Additional file 3** Known groups: No PU at baseline vs category 2 PU at baseline

| PU-QOL-P scales (n items) | Sample size | | Mean | SD | Mean | SD | P value* | Mean difference | CI for difference | Effect size |
|---|---|---|---|---|---|---|---|---|---|---|
| | Cat 2 PU at baseline | No PU baseline | Cat 2 PU at baseline | | No PU baseline | | | | | |
| Pain (12) | 18 | 151 | 13.342 | 21.520 | 10.908 | 20.679 | 0.639 | 2.434 | -7.79, 12.66 | 0.12 |
| Exudate (8) | 3 | 2 | 28.274 | 43.671 | 18.750 | 26.517 | 0.805 | 9.524 | -103.21, 122.26 | 0.25 |
| Odour (6) | 3 | 2 | 33.333 | 57.735 | 25.000 | 35.355 | 0.870 | 8.333 | -140.91, 157.57 | 0.16 |
| Sleep (7) | 15 | 143 | 13.571 | 31.258 | 8.851 | 19.661 | 0.408 | 4.720 | -6.52, 15.96 | 0.23 |
| Movement & mobility (9) | 15 | 132 | 17.287 | 34.338 | 13.177 | 28.387 | 0.604 | 4.110 | -11.52, 19.74 | 0.14 |
| Daily activities (6) | 15 | 134 | 16.111 | 31.255 | 7.152 | 20.055 | 0.126 | 8.959 | -2.54, 20.46 | 0.42 |
| Malaise (5) | 12 | 124 | 11.667 | 30.101 | 7.950 | 22.254 | 0.594 | 3.717 | -10.04, 17.47 | 0.16 |
| Emotional well-being (15) | 17 | 145 | 7.010 | 20.179 | 5.733 | 16.825 | 0.772 | 1.277 | -7.43, 9.98 | 0.07 |
| Self-consciousness and appearance (7 items) | 18 | 145 | 2.381 | 10.102 | 2.716 | 10.931 | 0.902 | -0.335 | -5.69, 5.02 | -0.03 |

* independent t-test, 0.05 significance value

**Table** Known groups: Braden score: completely limited vs no/slight impairment

| PU-QOL-P scales (n items) | Sample size | | Mean | SD | Mean | SD | | | | |
|---|---|---|---|---|---|---|---|---|---|---|
| | Completely limited^ | No/slight impairment^ | Completely limited | | No/slight impairment | | P value | Mean difference | CI for difference | Effect size |
| Pain (12) | 31 | 164 | 16.50 | 27.628 | 24.65 | 24.724 | 0.100 | -8.157 | -17.890, 1.576 | -0.32 |
| Exudate (8) | 4 | 26 | 0.00 | 0.000 | 2.68 | 8.886 | 0.557 | -2.679 | -11.916, 6.559 | -0.32 |
| Odour (6) | 4 | 26 | 0.00 | 0.000 | 0.96 | 4.903 | 0.702 | -0.962 | -6.058, 4.135 | -0.21 |
| Sleep (7) | 28 | 149 | 21.30 | 33.397 | 20.28 | 27.745 | 0.864 | 1.017 | -10.645, 12.680 | 0.04 |
| Movement & mobility (9) | 20 | 146 | 49.38 | 44.632 | 28.72 | 33.020 | 0.013* | 20.651 | 4.378, 36.924 | 0.60 |
| Daily activities (6) | 15 | 134 | 35.56 | 43.240 | 14.14 | 26.505 | 0.007* | 21.414 | 6.065, 36.762 | 0.75 |
| Malaise (5) | 22 | 136 | 24.09 | 31.609 | 15.63 | 30.086 | 0.226 | 8.466 | -5.286, 22.218 | 0.28 |
| Emotional well-being (15) | 24 | 147 | 16.03 | 26.241 | 13.01 | 26.260 | 0.602 | 3.017 | -8.395, 14.429 | 0.12 |
| Self-consciousness and appearance (7 items) | 25 | 145 | 12.29 | 24.530 | 6.69 | 16.014 | 0.141 | 5.596 | -1.880, 13.072 | 0.32 |

* independent t-test, 0.05 significance value

^Braden scores were recoded to create a dichotomous variable: completely limited (values range 6-12) vs no/slight impairment (values range 18-23).

**Table** Known groups: Location: Torso only vs limb only

| PU-QOL-P scales (n items) | Sample size | | Mean | SD | Mean | SD | | | | |
|---|---|---|---|---|---|---|---|---|---|---|
| | Limb | Torso | Limb | | Torso | | P value* | Mean difference | CI for difference | Effect size |
| Pain (12) | 29 | 96 | 23.55 | 25.551 | 25.10 | 25.321 | 0.773 | -1.550 | -12.19, 9.09 | -0.06 |
| Exudate (8) | 8 | 46 | 1.56 | 4.419 | 5.28 | 14.982 | 0.492 | -3.720 | -14.51, 7.07 | -0.27 |
| Odour (6) | 8 | 46 | .00 | .00 | .72 | 3.859 | 0.601 | -0.720 | -3.48, 2.04 | -0.20 |
| Sleep (7) | 25 | 89 | 26.90 | 34.537 | 23.53 | 31.505 | 0.644 | 3.370 | -11.06, 17.80 | 0.10 |
| Movement & mobility (9) | 22 | 74 | 35.32 | 41.949 | 38.57 | 35.930 | 0.721 | -3.250 | -21.26, 14.76 | -0.09 |
| Daily activities (6) | 21 | 77 | 22.62 | 37.652 | 20.28 | 31.701 | 0.774 | 2.340 | -13.80, 18.48 | 0.07 |
| Malaise (5) | 19 | 71 | 24.74 | 38.350 | 21.62 | 34.297 | 0.732 | 3.120 | -14.93, 21.17 | 0.09 |
| Emotional well-being (15) | 24 | 85 | 20.76 | 34.829 | 12.07 | 23.227 | 0.154 | 8.690 | -3.30, 20.68 | 0.33 |
| Self-consciousness and appearance (7 items) | 23 | 87 | 18.17 | 30.965 | 6.73 | 16.352 | 0.017 | 11.440 | 2.05, 20.83 | 0.57 |

* independent t-test, 0.05 significance value
