# Supplementary material for: A patient-reported pressure ulcer health-related quality of life instrument for use in prevention trials (PU-QOL-P): psychometric evaluation
Source: Health Qual Life Outcomes. 2018 Dec 10;16:227. doi: 10.1186/s12955-018-1049-x (PMC6288857; doi:10.1186/s12955-018-1049-x)
Supplement: Supplementary file 4 — Modified scales post hoc analysis (PDF 93 kb) [file 12955_2018_1049_MOESM4_ESM.pdf]

Modified scales post hoc analysis

Table 2 PU-QOL-R scale level analyses - Reliability and scaling assumptions: Validity within-scale analysis

PU-QOL-P scales (n items)	Internal consistency		Mean inter-item correlation	Inter-item correlation^	Scaling Assumptions -Corrected ITC^
	n	Cronbach's alpha			
Daily activities (5)	189	0.959	0.789	0.644 – 0.901	0.750 – 0.927
Malaise (4)	224	0.921	0.743	0.634 – 0.849	0.715 – 0.879
Emotional well-being (15)	215	0.971	0.689	0.513 – 0.899	0.682 – 0.878
Self-consciousness and appearance (7)	295	0.914	0.645	0.502 – 0.795	0.681 – 0.812

SEM standard error mean; *IIC* inter-item correlation; *ITC* item-total correlation (corrected for overlap)

^Range

Table Spearman's rho correlations between PU-QOL-R and SF12 scales (convergent validity)

Scale (n items)	Physical function (2)	Role physical (2)	Bodily pain (1)	General health (1)	Vitality (1)	Social function (1)	Role emotional (2)	Mental health (2)
Daily activities (5)	0.120*	0.130**	0.224**	0.184**	0.085	0.047	0.278**	0.266**
Malaise (4)	0.046	0.004	0.253**	0.244**	0.039	0.062	0.312**	0.312**
Emotional well-being (15)	0.044	0.041	0.227**	0.184**	0.003	0.023	0.343**	0.294**
Self-consciousness and appearance (7 items)	0.030	0.048	0.216**	0.137**	0.039	0.054	0.246**	0.230**

*Correlation is significant at the 0.05 level (2-tailed).

**Correlation is significant at the 0.01 level (2-tailed).

Table PU-QOL-P Responsiveness over time: PU at baseline (visit 0) compared to no PU 30 days post-treatment end (visit 30)

PU-QOL-P scales (n items)	N	Baseline scores	Visit30 scores	Mean change	Effect Size	CI	P value
		Mean (SD)	Mean (SD)				
Daily activities (5)	22	22.20 (36.185)	11.5530 (27.209)	10.644	0.33	-7.695, 28.983	0.241
Malaise (4)	21	23.81 (39.903)	4.7619 (16.991)	19.048	0.62	3.820, 34.275	0.017
Emotional well-being (15)	32	14.22 (26.162)	0.5208 (2.946)	13.700	0.74	4.251, 23.149	0.006
Self-consciousness and appearance (7)	31	9.06 (19.279)	0.0000 (0.000)	9.063	0.66	1.992, 16.134	0.014
